# Sensory Processing in the Dorsolateral Striatum: The Contribution of Thalamostriatal Pathways

**DOI:** 10.3389/fnsys.2017.00053

**Published:** 2017-07-25

**Authors:** Kevin D. Alloway, Jared B. Smith, Todd M. Mowery, Glenn D. R. Watson

**Affiliations:** ^1^Neural and Behavioral Sciences, Center for Neural Engineering, Pennsylvania State University University Park, PA, United States; ^2^Molecular Neurobiology Laboratory, The Salk Institute for Biological Studies La Jolla, CA, United States; ^3^Center for Neural Science, New York University New York, NY, United States; ^4^Department of Psychology and Neuroscience, Duke University Durham, NC, United States

**Keywords:** corticostriatal, intralaminar complex, Parkinson disease, POm nucleus, sensorimotor, superior colliculus, thalamus, zona incerta

## Abstract

The dorsal striatum has two functionally-defined subdivisions: a dorsomedial striatum (DMS) region involved in mediating goal-directed behaviors that require conscious effort, and a dorsolateral striatum (DLS) region involved in the execution of habitual behaviors in a familiar sensory context. Consistent with its presumed role in forming stimulus-response (S-R) associations, neurons in DLS receive massive inputs from sensorimotor cortex and are responsive to both active and passive sensory stimulation. While several studies have established that corticostriatal inputs contribute to the stimulus-induced responses observed in the DLS, there is growing awareness that the thalamus has a significant role in conveying sensory-related information to DLS and other parts of the striatum. The thalamostriatal projections to DLS originate mainly from the caudal intralaminar region, which contains the parafascicular (Pf) nucleus, and from higher-order thalamic nuclei such as the medial part of the posterior (POm) nucleus. Based on recent findings, we hypothesize that the thalamostriatal projections from these two regions exert opposing influences on the expression of behavioral habits. This article reviews the subcortical circuits that regulate the transmission of sensory information through these thalamostriatal projection systems, and describes the evidence that indicates these circuits could be manipulated to ameliorate the symptoms of Parkinson’s disease (PD) and related neurological disorders.

In the 19th century, William James wrote that “Any sequence of mental action which has been frequently repeated tends to perpetuate itself; so that we find ourselves automatically prompted to think, feel, or do what we have been before accustomed to think, feel, or do under like circumstances, without any consciously formed purpose, or anticipation of results” (James, 1887). This statement identifies the critical features that distinguish habitual behaviors from those that require conscious effort. While “consciously formed” actions are aimed at acquiring specific goals or “anticipated results”, habits are “automatically prompted” if they are “frequently repeated” in a similar context or “like circumstances”.

More than 125 years later, the neural basis for this behavioral dichotomy is only partially understood. Substantial evidence indicates that the dorsomedial striatum (DMS) is involved with encoding the relationship between conscious actions and the value of the outcomes produced by those actions, which are classified as goal-directed behaviors. By comparison, the dorsolateral striatum (DLS) is necessary for the expression of behavioral habits that are automatically evoked in a familiar context (Yin and Knowlton, 2006; Balleine et al., 2009; Balleine and O’Doherty, 2010; Devan et al., 2011; Seger and Spiering, 2011). In fact, the strong relationship between sensory context and habitual behavior has led to the view that DLS is needed to form stimulus-response (S-R) associations (Graybiel, 2008; Devan et al., 2011).

Substantial evidence indicates that DLS neurons respond to sensory stimulation, but the neural basis for these responses is in dispute. While some results suggest that DLS receives its sensory inputs entirely from sensorimotor cortex (Pidoux et al., 2011; Reig and Silberberg, 2014; Wilson, 2014), direct comparisons of stimulus-evoked discharges in DLS and primary somatosensory cortex (SI) indicate that other brain regions must be involved in shaping the sensory responses in DLS (Mowery et al., 2011; Smith et al., 2012; Alloway et al., 2014). In fact, the dorsal striatum is innervated by several thalamic nuclei that process sensory information, but the functions of most thalamostriatal systems remain unclear. While much is known about the centromedian-parafascicular (CM-Pf) complex and its functional role (Ding et al., 2010; Galvan and Smith, 2011; Smith et al., 2014), the potential influence exerted by posteromedial nucleus (POm) on striatal responsiveness is just beginning to be recognized (Watson et al., 2015).

After reviewing the evidence indicating that the striatum receives sensory inputs from both cortex and thalamus, we will describe the experimental results implicating the POm in transmitting sensory information to DLS. As part of this, we will describe how POm is regulated by a multi-synaptic circuit in which the superior colliculus activates the zona incerta (ZI), which sends GABAergic projections to POm. This feedforward inhibitory circuit is functionally significant because deep brain stimulation (DBS) in ZI produces beneficial effects in the treatment of Parkinson’s disease (PD) and related movement disorders (Sun et al., 2008; Caire et al., 2013; Garcia-Garcia et al., 2016). To the extent that the sensory information conveyed from POm facilitates the behavioral functions of the DLS, the therapeutic effects of ZI stimulation in humans could be mediated by disrupting the inhibitory influence exerted by ZI on the human homolog of POm.

## Dual Control Systems in the Striatum

One of the first indications that the striatum processes sensory information came from studies that used a water maze to compare different neural systems in remembering visual stimuli and spatial locations. In one study (Packard and McGaugh, 1992), two partially-submerged balls labeled by horizontal or vertical strips were used to mark a hidden platform (correct choice) or a thin pedestal (incorrect choice). In the spatial task, the rat learned to find the escape platform by swimming to the same spatial location regardless of the pattern on the target ball. In the S-R version of the task, the platform’s location was varied but it could be located by swimming to the ball with the same pattern. Fornix lesions impaired performance on the spatial task, but did not impair the S-R habit. Conversely, striatal lesions impaired performance on the S-R task, but not on the spatial task.

Subsequent work showed that lesions of DLS alone were sufficient to establish its role in stimulus-controlled behavior. Using a similar water maze task, rats with bilateral DMS lesions still swam to different locations marked by the same visual cue. By contrast, rats with bilateral DLS lesions always swam to the same spatial location (Devan and White, 1999; Devan et al., 1999).

### Goal-Directed and Stimulus-Controlled Behaviors

Studies that analyzed differences between goal-directed and stimulus-controlled behaviors have been instrumental in identifying the behavioral functions of the striatum. These behavioral categories are often distinguished by operant conditioning paradigms that vary either the value of a reward or its probability of delivery. If a rat is trained to press a lever for food that is subsequently paired with an aversive agent, this devaluation in reward reduces lever-pressing behavior (Adams and Dickinson, 1981; Dickinson, 1985). Consistent with this example, goal-directed behaviors are sensitive to changes in reward value. By contrast, the defining characteristic of a stimulus-controlled habit is its resistance to reward devaluation (Adams, 1982; Dickinson, 1985).

Most behaviors start as goal-directed, but they can be transformed into a behavioral habit by prolonged overtraining or by reducing the predictability of the reward (Dickinson et al., 1983). When goal-directed behaviors are repeatedly expressed in the same sensory context, their transformation into a behavioral habit has several adaptive advantages. In addition to increased efficiency and other improvements in skill that normally ensue with repeated performance, habitual expression of a behavior in a specific context allows valuable neural resources to be devoted to other cognitive tasks that are more demanding (Graybiel, 2008). Learning to drive a car, for example, requires focused attention during the initial training stages. With repeated practice, however, acquisition of good driving habits allows a driver to engage in thoughtful conversation while navigating a well-known route on “auto-pilot”.

### Functional Roles of DMS and DLS

Selective inactivation of DMS or DLS indicates that these brain regions are differentially involved in the production of goal-directed and habitual behaviors (Redgrave et al., 2010). After a behavioral habit is acquired, DLS inactivation causes habitual activity to be replaced by goal-directed actions that are mediated by the intact DMS (Yin et al., 2004; Yin and Knowlton, 2006). Conversely, when DMS is inactivated, goal-directed behaviors are replaced by motor habits because the DLS remains intact (Yin et al., 2005).

**Figure 1:**
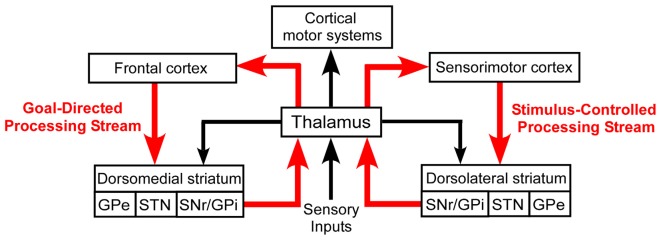
Basal ganglia processing streams involved in the expression of goal-directed and stimulus-controlled behaviors. According to the prevailing view, virtually all sensory information is transmitted to the striatum by a series of thalamocortical and corticostriatal connections. The thalamus also sends direct projections to the striatum, but its functional influence is poorly understood.

Despite their differential involvement in goal-directed and stimulus-controlled behaviors, both DMS and DLS are considered to be cortically driven. As shown in Figure 1, goal-directed behaviors are usually associated with interactions between prefrontal cortex and DMS, while habitual behaviors are generally considered as being dependent on projections from sensorimotor cortex to the DLS (Yin et al., 2004, 2005; Yin and Knowlton, 2006; Redgrave et al., 2010; Hernández et al., 2015). In this scheme, the thalamus is part of a multi-synaptic loop that integrates output signals from both the dorsomedial and dorsolateral processing streams of the basal ganglia and, in turn, transmits this processed information to motor cortex.

The thalamus also sends projections to both the DMS and DLS. Thalamostriatal projections are known to be derived from a phylogenetically-old system that has a role in behavioral selection (McHaffie et al., 2005), but the precise nature of this role and the capacity of thalamostriatal systems for transmitting sensory-specific information has not been fully elucidated. A growing body of evidence, however, indicates that POm transmits sensory information to DLS, presumably to facilitate the expression of habitual behavior.

## DLS Involvement in Sensorimotor Habits

Chronic recordings of DLS neurons across successive training sessions have led to several insights about the possible mechanisms by which goal-directed behaviors are transformed into behavioral habits that are encoded in DLS. In one study, DLS responses and forelimb EMG activity were recorded simultaneously as a rat learned to press a lever for a reward in a specific sensory context (Carelli et al., 1997). Initially, the forelimb-sensitive neurons in DLS were activated prior to each cued lever press, but these neuronal responses gradually subsided as training progressed. The neurons continued to respond, however, during other forelimb movements or in response to external cutaneous stimulation. The disparity between conditioned-induced suppression of DLS activity and the persistent responsiveness to other forms of active or passive sensory stimulation suggests that the emergence of habitual behavior is associated with neuroplasticity in specific neural circuits. Furthermore, the persistence of DLS responses to some forms of sensory stimulation suggests that sensory information can be transmitted to DLS by routes that are not altered by the acquisition of a behavioral habit.

### Striatal Encoding of Sequence Learning

Several chronic recording studies indicate that DLS activity is altered during the acquisition of habits that consist of specific behavioral sequences. In two related studies, rats were trained to navigate a T-maze by turning right or left according to a cue that signaled the location of a reward (Jog et al., 1999; Barnes et al., 2005). Initially, DLS neurons discharged continuously as the rat proceeded through the maze, but with progressive training the DLS activity was transformed so that neurons discharged at the start and end of each trial but not during the middle part of the task. This “beginning-and-end bracketing pattern” in DLS could represent the neural correlate for “chunking” multiple behavioral elements into a specific sequence (Graybiel, 1998; Smith and Graybiel, 2014). In fact, behavioral lesion experiments have confirmed that DLS is necessary for encoding behavioral habits that consist of specific sequences of lever presses (Yin, 2010).

The expression of behavioral habits in a familiar context has prompted the belief that DLS is needed to form S-R associations. According to this view, habits that consist of a specific sequence of behavioral elements could be encoded by a chain of S-R associations in DLS, in which the sensorimotor elements of each behavioral action serve as stimuli for evoking the next behavioral response. But this possibility seems to be contradicted by the task-bracketing pattern of neural activity observed in DLS (Jog et al., 1999; Barnes et al., 2005; Smith and Graybiel, 2014).

Suppression of DLS activity during the interval between the start and end of a well-learned behavioral sequence suggests that each element in the behavioral sequence is not correlated with a specific S-R association in DLS. While stimulus-induced information might be transmitted faithfully to DLS during the initial stages of behavioral acquisition, repetitive training appears to recruit circuit mechanisms that alter DLS activity as a behavioral sequence becomes habitual, thereby gating certain sensory inputs so that only the start and end of the behavioral habit are correlated with DLS responsiveness (Smith and Graybiel, 2014). Relevant to this point, a similar task-bracketing pattern also appears in infralimbic cortex, which suggests that similar changes in striatal and cortical activity are essential for the consolidation of a behavioral habit (Smith and Graybiel, 2014).

Other chronic recording studies have confirmed that striatal neurons can encode the beginning, the end, or both the start and finish of a well-learned behavioral sequence (Jin and Costa, 2010). But these studies have also revealed additional types of striatal neurons, including those that show sustained activity throughout an entire behavioral sequence (Jin et al., 2014; Jin and Costa, 2015). In experiments in which mice habitually performed bar presses in rapid succession, many striatal neurons were continuously active throughout the entire bar-press sequence, and modulations in their rate of activity were correlated with the execution of individual bar presses (Jin et al., 2014). This suggests that subpopulations of striatal neurons encode sequences of individual motor acts by discharging at an increased rate as each motor element is expressed. By contrast, when primary motor cortex (MI) was recorded during these motor sequences, half of the neurons signaled the start or end of the sequence, but very few of them displayed activity changes that were correlated with individual motor elements (Jin et al., 2014). For those striatal neurons that encode each motor action, this pattern must depend on circuit mechanisms that extend beyond the corticostriatal signals sent from MI cortex.

While it is clear that sensorimotor information is transmitted to DLS during consolidation of a behavioral habit, there is considerable variation in the dynamics of the responses recorded in different subpopulations of DLS neurons during this learning process. While some changes undoubtedly involve neuroplasticity, the exact mechanisms are unclear. Dopamine signals have been identified as mediators in striatal learning (Surmeier et al., 2007; Shen et al., 2008), and some have suggested that the dopamine signals involved in reinforcing striatal detection of a sensory stimulus could selectively potentiate those corticostriatal inputs that transmit sensory-relevant signals (Donahue and Kreitzer, 2015). But the possibility that thalamostriatal inputs could contribute to the DLS responses that enable a behavioral habit has never been considered.

## Innate Behaviors Regulated by DLS

Relevant to its role in mediating well-learned behaviors, the DLS is also involved in the expression of species-specific behaviors that are considered innate. In rodents, behaviors such as grooming and exploratory whisking do not depend on acquisition of a reward, but are characterized by highly-repetitive, sensory-guided stereotyped movements that are usually emitted in specific contexts.

### Behavioral Grooming

Behavioral lesion experiments indicate that DLS has a role in grooming behavior, which consists of stereotyped head and limb movements that follow a predictable sequence known as a “syntactic chain”. Bilateral lesions at specific sites in the DLS disrupt grooming behavior by altering the sequence and duration of individual behavioral elements, or by omitting certain elements altogether even though the ability to make a specific grooming movement remains intact (Cromwell and Berridge, 1996).

The DLS lesions that affect grooming behavior are located at sites that receive dense inputs from the forelimb representations in the SI and MI cortical areas (Cromwell and Berridge, 1996; Brown et al., 1998; Hoffer and Alloway, 2001; Hoover et al., 2003). Neurons at these DLS sites are active during grooming behavior, and while some neuronal activity is correlated with specific grooming movements, most neurons appear to be encoding the repetition of a complete syntactic grooming chain (Aldridge and Berridge, 1998). Lesions in the MI regions that project to DLS have no effect on the pattern of grooming behavior (Berridge and Whishaw, 1992), and this suggests that another brain region sends DLS the information needed to maintain the syntactic chain. Likewise, MI areas that project to DLS are needed during the acquisition of a well-learned motor skill such as bar-pressing, but are not needed once the behavior has been consolidated (Kawai et al., 2015). This suggests that other inputs to the striatum are responsible for the expression of a well-learned motor habit once it has been consolidated.

### Exploratory Whisking

Rats and mice actively sweep their whiskers to acquire sensory information about local surroundings and objects during behavioral exploration. Active whisking consists of highly-repetitive, rhythmic excursions of the whiskers, usually in the range of 5–15 Hz (Welker, 1964; Berg and Kleinfeld, 2003). Prior to contacting an object, whisking is bilaterally coordinated so that whisker movements on both sides of the head are relatively symmetric in terms of their frequency and amplitude (Gao et al., 2001; Mitchinson et al., 2007).

Substantial evidence implicates DLS with a role in exploratory whisking. The caudal DLS receives dense overlapping projections from several cortical whisker regions (Brown et al., 1998; Alloway et al., 1999, 2006, 2009; Hoffer and Alloway, 2001), and DLS neurons display rhythmic discharge patterns during exploratory whisking in the awake behaving rat (Carelli and West, 1991). Furthermore, in the lightly-anesthetized rat, neurons in caudal DLS display high-fidelity neuronal discharges in response to repetitive whisker deflections (Mowery et al., 2011; Smith et al., 2012).

Consistent with other habitual behaviors, exploratory whisking does not depend on a rewarded outcome, and certain aspects of exploratory whisking are characterized by stereotyped patterns of whisker excursions that resemble S-R associations. For example, when whiskers on one side contact an external stimulus during active whisking, the contralateral whiskers automatically exhibit larger sweeping movements while the contacted whiskers display smaller excursions (Sachdev et al., 2003; Mitchinson et al., 2007).

Exploratory whisking is characterized by a series of epochs in which whisks are repetitively emitted at a specific frequency and amplitude for 1–2 s before another whisking epoch is emitted at a new frequency and amplitude (Berg and Kleinfeld, 2003). This pattern suggests that whisking behavior consists of a series of behavioral segments that are “chunked” together. Whether these frequency-defined epochs follow a specific syntactic chain is unknown because no study has examined whether DLS lesions alter either the kinematic or syntactic features of whisking behavior.

The repetitive stereotyped nature of whisking, along with the fact that DLS neurons respond to both active and passive whisker movements, has prompted several investigators to use the whisker system to analyze sensory processing in DLS (West, 1998; Mowery et al., 2011; Pidoux et al., 2011; Reig and Silberberg, 2014). The peripheral whiskers are densely innervated and, consequently, a major advantage of this model is that the whisker representations in thalamus, cortex and DLS are disproportionately larger than other somatic representations in these regions (Chapin and Lin, 1984; Fabri and Burton, 1991; Brown et al., 1998; Alloway et al., 2003, 2006).

## Neural Circuits for Transmitting Sensory Information to DLS

**Figure 2:**
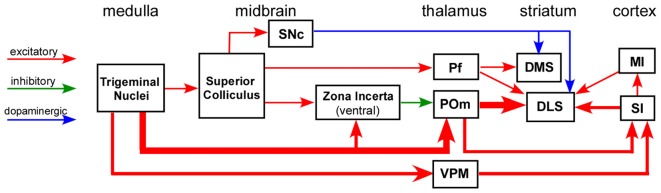
Circuit diagram illustrating multiple neuronal pathways for conveying sensory inputs to the striatum. The thick afferent and efferent connections of the POm indicate the most direct route for transmitting somesthetic information to the DLS. Abbreviations: DMS, dorsomedial striatum; DLS, dorsolateral striatum; MI, primary motor cortex; Pf, parafascicular nucleus; POm, posteromedial nucleus; SI, primary somatosensory cortex; SNc, substantia nigra pars compacta; VPM, ventroposteromedial nucleus. Adapted from Watson et al. (2015).

Substantial effort has been devoted to elucidating the input-output connections of the striatum, including the neuronal routes that convey sensory information to DLS. While many studies have characterized the topography and other aspects of corticostriatal connectivity, far fewer have been devoted to elucidating the subcortical circuits that influence striatal processing. Nonetheless, as indicated by Figure 2, several neuronal circuits are capable of transmitting sensory-related information to the striatum without involving the cerebral cortex.

### Corticostriatal Projections

The DLS receives a massive number of divergent and convergent projections from sensorimotor cortex (McGeorge and Faull, 1989; Brown et al., 1998; Wright et al., 1999; Hoffer and Alloway, 2001; Hintiryan et al., 2016). The combined influence of multiple divergent projections from SI, SII, MI and related cortical regions is assumed to be responsible for the fact that DLS has a discernible, but relatively crude somatotopic organization (Carelli and West, 1991; Brown et al., 1998).

Several anatomical studies indicate that corticostriatal projections are characterized by substantial amounts of divergence. Tracer deposits in the SI and MI limb of primates have consistently revealed a “one-to-many” corticostriatal projection pattern (Flaherty and Graybiel, 1991, 1994). Similarly, in rats, focal tracer deposits in a single cortical column of the SI barrel field produced separate and distinct patches of labeled terminals in the DLS neuropil (Alloway et al., 1998).

Corticostriatal divergence has also been demonstrated with the 2-deoxyglucose (2-DG) labeling technique (Brown, 1992). Stimulation of different body parts (e.g., trunk or limbs) reveals a crude somatotopic map that resembles the overlying cortical topography, but 2-DG patches representing different body sites are often juxtaposed in different combinations. This “combinatorial map” suggests that every DLS site could receive converging inputs from separate cortical regions that are activated during a behavioral activity. Relevant to this result, DLS recordings have shown that adjacent neurons often respond to stimulation of separate body parts (Carelli and West, 1991).

The complexity of the functional topography of DLS is further emphasized by the presence of substantial corticostriatal convergence. Corresponding somatotopic representations in SI, SII and MI project to overlapping parts of DLS (Flaherty and Graybiel, 1993, 1995; Alloway et al., 2000; Hoffer and Alloway, 2001), and ultrastructural analysis indicates that projections from rat SI and MI whisker regions converge on individual DLS neurons (Ramanathan et al., 2002). This is significant because corticostriatal axons have few collaterals, they traverse the striatal neuropil along a predominantly straight path, and each axon contributes few synapses to each medium spiny neuron (MSN) that it contacts (Wilson, 1995; Kincaid et al., 1998). These findings suggest that DLS discharges must depend on synchronous activation of multiple cortical areas that send convergent projections to the DLS.

The DLS also receives inputs from sensorimotor cortex in the contralateral hemisphere (Wilson, 1987; Wright et al., 2001; Reiner et al., 2003; Wu et al., 2009). Consistent with these intra-telencephalic projections (Reiner et al., 2003), small unilateral tracer deposits in DLS have revealed mirror-image distributions of neuronal labeling throughout multiple sensorimotor cortical areas in both hemispheres (Alloway et al., 2006). Hence, the large number of cortical sites in both hemispheres that send converging projections to DLS underscores the number of different combinations of cortical neurons that can cooperate with each other to activate DLS.

### Thalamostriatal Projections

Thalamostriatal projections originate from several nuclei, but not all of them process sensory information. The thalamic nuclei that are principally involved in conveying sensory-related information to the striatum include POm, the CM/Pf complex and other intralaminar nuclei, and the lateral posterior (LP) and lateral dorsal (LD) nuclei. While the lateral Pf nucleus in rodents represents the homolog of the primate CM nucleus (Galvan and Smith, 2011), the rodent POm is considered to be the homolog of the anterior part of the primate pulvinar (Butler and Hodos, 2005). Both Pf and POm send projections to the sensorimotor striatum (i.e., DLS in rats and putamen in primates).

Most thalamostriatal research has focused on the CM/Pf complex because it contains more thalamostriatal neurons than any other region. Consistent with evidence suggesting that tecto-thalamic pathways represent a phylogenetically-old system for transmitting sensory information to the basal ganglia (McHaffie et al., 2005), the CM/Pf complex receives a large number of multimodal sensory inputs from the intermediate and deep layers of the superior colliculus (Krout et al., 2001). The CM/Pf complex is also notable for having relatively weak connections with the cerebral cortex (Galvan and Smith, 2011).

The LP and LD nuclei receive sensory inputs that are largely visual in nature. The LP nucleus receives inputs from both the upper collicular layers and from several visual cortical areas (Donnelly et al., 1983; Sugita et al., 1983; Abramson and Chalupa, 1988; Lane et al., 1993, 1997; Roth et al., 2016). By comparison, the LD nucleus receives visual-related input from the pretectal area, the intermediate layers of the superior colliculus, and the occipital cortex (Arango and Scalia, 1978; Robertson, 1983; Thompson and Robertson, 1987).

The POm nucleus is distinct from LP, LD and the CM/Pf complex in that it receives very few inputs from the superior colliculus (Roger and Cadusseau, 1984). Instead, POm is dominated by ascending and descending somesthetic inputs that originate, respectively, from the trigeminal nuclei and from multiple sensorimotor cortical areas (Chiaia et al., 1991a,b; Diamond et al., 1992; Williams et al., 1994; Veinante et al., 2000; Alloway et al., 2003; Killackey and Sherman, 2003).

In contrast to the CM/Pf complex, which has sparse connections with cortex, the POm, LP and LD nuclei are strongly connected with specific cortical areas (Galvan and Smith, 2011; Smith et al., 2014). Furthermore, these thalamic nuclei and their respective cortical targets send converging projections to specific striatal regions (Groenewegen and Berendse, 1994). The convergence of thalamostriatal and corticostriatal projections from sites that are reciprocally-connected suggests an obvious circuit mechanism for promoting cooperative activation of their common targets in the striatum.

Rodent studies indicate that thalamostriatal projections from LP, LD, POm and Pf differ significantly in terms of the striatal regions that they innervate. While Pf projections terminate throughout the dorsal striatum (Alloway et al., 2014), the LD, LP and POm nuclei innervate more restricted striatal territories. The LD nucleus is reciprocally connected with the agranular motor and posterior parietal cortices (Chandler et al., 1992; Reep and Corwin, 1999), and it projects densely to the dorsal periphery of the rat striatum (Kamishina et al., 2008). The LP nucleus is connected with the medial agranular and posterior parietal cortices (Reep and Corwin, 2009), and it innervates the dorsal central striatum and a narrow part of the rostral DLS (Kamishina et al., 2008). In contrast to these thalamic nuclei, POm is the only thalamic nucleus that innervates DLS without projecting to any other striatal region (Deschênes et al., 1995, 1996; Smith et al., 2012; Alloway et al., 2014).

## Comparisons of Pf and POm Neurons and their Connections

The DLS receives projections from both Pf and POm, but these nuclei differ substantially in terms of their neuronal morphology. Neurons in Pf have an oval soma from which a few poorly-branched dendrites extend over distances ranging between 0.5 mm and 1.5 mm (Hazlett et al., 1976; Deschênes et al., 1996; Lacey et al., 2007). These relatively long, poorly-branched dendrites are considered ideal for integrating different types of information from a variety of sources (Deschênes et al., 1995).

By contrast, POm neurons have highly-branched dendritic arbors that resemble the classic description of bushy relay neurons in ventral posteromedial nucleus (VPM) and other thalamic relay nuclei (Deschênes et al., 1995; Ohno et al., 2012). These bushy dendrites extend 300–400 μm from the soma in all directions, thereby endowing POm neurons with a large surface area for receiving many synaptic contacts within a confined space. In addition to receiving ascending projections from the spinal trigeminal nucleus (Chiaia et al., 1991a; Williams et al., 1994; Veinante et al., 2000), POm also receives descending corticothalamic projections from SI cortex that form large synaptic terminals known as “drivers”, as well as smaller synaptic terminals from MI (Sherman and Guillery, 1998; Alloway et al., 2008). Ultrastructural analysis demonstrates that these trigeminothalamic and corticothalamic projections converge synaptically onto common neuronal targets in POm (Groh et al., 2013). Consistent with this finding, POm neurons are most likely to discharge when they receive near-coincident inputs from these converging projections (Groh et al., 2013).

### Differential Projection Patterns of Pf and POm Neurons

Axonal projections from Pf and POm differ in several important respects. Axons emerging from Pf pass through the reticular nucleus, where they emit a few sparsely-branched collaterals before proceeding towards the basal ganglia to provide collateral innervation to the globus pallidus (GP), subthalamic nucleus (STN) and entopeduncular nucleus before diverging into multiple branches that terminate throughout the dorsal striatum, including its medial and lateral sectors (Deschênes et al., 1996; Lacey et al., 2007; Alloway et al., 2014). Although Pf sends some projections to motor cortex, the density of these cortical terminals is significantly lower than in the striatum (Galvan and Smith, 2011).

By contrast, the caudal half of POm projects to the caudal part of DLS, but these connections represent collaterals of the main axon, which innervates sensorimotor cortex (Deschênes et al., 1995; Ohno et al., 2012). Furthermore, whereas Pf projects sparsely to sensorimotor cortex, the projections from caudal POm terminate densely in SI, SII and to a lesser extent in MI (Lu and Lin, 1993; Wimmer et al., 2010; Viaene et al., 2011; Ohno et al., 2012; Smith et al., 2012). In fact, the thalamocortical projections from POm terminate most densely in layer Va (Wimmer et al., 2010; Smith et al., 2012), which contains most of the corticostriatal neurons that project to DLS (Reiner et al., 2003). Significantly, the apical dendrites of layer V corticostriatal neurons receive very few synaptic contacts from the ventroposterior nuclei (i.e., VPM and VPL), even though they extend into layer IV, which is densely innervated by these thalamic nuclei (Hersch and White, 1982). Hence, when compared to the classic lemniscal circuit for transmitting somesthetic information to SI cortex, differences in the density and cellular distribution of synaptic contacts suggest that POm is probably more efficacious in activating corticostriatal neurons than the thalamocortical projections from VPM or VPL.

**Figure 3:**
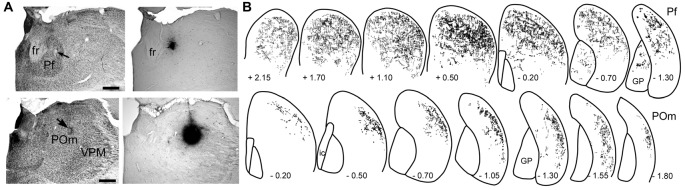
Patterns of striatal innervation revealed by placing an anterograde tracer in Pf (top) or POm (bottom). **(A)** Photomicrographs of adjacent sections depict cytoarchitecture and biotinylated dextran amine (BDA) deposits in Pf or POm of two different rats. **(B)** Rostrocaudal series showing spatial distribution of BDA-labeled axonal varicosities in the striatum. Each series based on superimposing reconstructions from three rats that received similar BDA injections in Pf or POm. Numbers indicate distance (mm) from bregma. Scale bars: 500 microns in **(A)**. Abbreviations: fr, fasciculus retroflexus; GP, globus pallidus; VPM, ventroposteromedial nucleus. Adapted from Alloway et al. (2014).

As shown in Figure 3, the striatal territories innervated by Pf and POm are significantly different. Although Pf is considerably smaller than POm, it provides extensive innervation throughout the medial and lateral parts of the dorsal striatum. By comparison, thalamostriatal projections from caudal POm terminate in DLS, especially its caudal half, but do not innervate any other striatal region (Ohno et al., 2012; Alloway et al., 2014). Consistent with these different projection patterns, the axonal arbors of Pf branch much more extensively than those originating from POm (Deschênes et al., 1995; Lacey et al., 2007).

**Figure 4:**
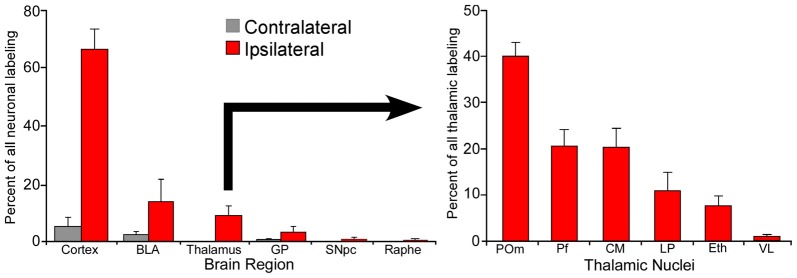
Proportion of labeled neurons in different brain regions after injecting a retrograde tracer into the right DLS of seven rats. More than 70% of the projections originate from the two cortical hemispheres, nearly 20% from the basolateral amygdala (BLA), and 10% from the thalamus. Among thalamic neurons that project to DLS, 40% reside in the medial part of the posterior nucleus (POm). Adapted from Smith et al. (2012).

Although individual neurons in Pf contribute more axonal terminals to the striatum, POm is much larger than Pf and contains many more neurons that project to the DLS. In fact, as illustrated by Figure 4, retrograde tracer injections in DLS revealed that POm contains the largest number of thalamic neurons that project to DLS (Smith et al., 2012). These numerical differences, along with the fact that POm receives direct trigeminal inputs and is strongly connected with corticostriatal neurons in SI, suggest that POm is likely to be an important source of the somesthetic information transmitted to DLS.

### Synaptic Connectivity of Pf and POm Neurons

Thalamostriatal projections from Pf and other intralaminar nuclei form axo-dendritic synapses on both MSNs and on large cholinergic interneurons, which display high rates of spontaneous activity and are often called tonically-active neurons (Dube et al., 1988; Meredith and Wouterlood, 1990; Lapper and Bolam, 1992; Raju et al., 2006; Lacey et al., 2007; Smith et al., 2014). Innervation of cholinergic interneurons by CM/Pf is noteworthy because electrical stimulation of CM/Pf evokes a burst of activity in these cells that is followed by a sequential cascade of striatal events that differentially alter the influence of corticostriatal projections on the MSNs associated with the direct or indirect pathways (Ding et al., 2010; Thorn and Graybiel, 2010).

No study has characterized the synaptic contacts of the thalamostriatal projections from POm. Presumably, if POm projections have the same synaptic pattern reported for other non-CM/Pf nuclei (Dube et al., 1988; Raju et al., 2006; Lacey et al., 2007; Smith et al., 2014), axonal projections from POm should contact the spines of MSN dendrites. If true, this would be significant because corticostriatal terminals also form synaptic contacts on the dendritic spines of MSNs (Dube et al., 1988; Wright et al., 1999; Raju et al., 2008). Given that individual neurons in POm send collateral projections to both DLS and corticostriatal neurons in layer Va (Deschênes et al., 1996; Smith et al., 2012), this putative synaptic configuration should increase the likelihood that the dendritic spines of DLS neurons receive near-coincident excitatory inputs, first from POm and then from the corticostriatal neurons activated by POm.

## Striatal Responses to Sensory Stimulation

The first experiments that recorded sensory responses in the striatum were done on cats in the 1960s (Albe-Fessard et al., 1960; Sedgwick and Williams, 1967). Since then, numerous studies have shown that striatal neurons in primates, cats and rodents can respond to visual, auditory, or somesthetic stimulation (Schneider and Lidsky, 1981; Crutcher and DeLong, 1984; DeLong et al., 1984; Alexander and DeLong, 1985; Lidsky et al., 1985; Strecker et al., 1985; Hikosaka et al., 1989; Carelli and West, 1991; Bordi and LeDoux, 1992; Bordi et al., 1993; Carelli et al., 1997; West, 1998; Cromwell et al., 2007; Schulz et al., 2009; Mowery et al., 2011; Pidoux et al., 2011; Syed et al., 2011; Hawking and Gerdjikov, 2013; Znamenskiy and Zador, 2013; Sippy et al., 2015; Xiong et al., 2015). In fact, depending on their location, striatal neurons may integrate inputs from two or more sensory modalities (Krauthamer, 1979; Chudler et al., 1995; Nagy et al., 2006; Reig and Silberberg, 2014).

The striatum can be activated by different sensory modalities, but the latencies of the responses vary considerably depending on the modality of the stimulus. Somesthetic-induced responses in the striatum have latencies of 5–10 ms in rats (Mowery et al., 2011; Reig and Silberberg, 2014), and 10–20 ms in cats and primates (Schneider and Lidsky, 1981; DeLong et al., 1984). Striatal responses to auditory stimuli also have short latencies of 10–20 ms (Bordi and LeDoux, 1992; Bordi et al., 1993; Cromwell et al., 2007). By contrast, striatal responses to visual stimuli have much longer latencies, ranging from 100 ms to 150 ms (Schulz et al., 2009; Reig and Silberberg, 2014).

These latency differences suggest that auditory and somesthetic information are transmitted to the striatum across relatively short neuronal routes, whereas visual information must reach the striatum by longer multi-synaptic circuits. In fact, just as somesthetic information can be transmitted directly to DLS by thalamostriatal projections from caudal POm (Ohno et al., 2012; Smith et al., 2012; Alloway et al., 2014), some reports indicate that auditory information can be transmitted directly from the medial geniculate nucleus to the ventral DLS (Ryugo and Killackey, 1974; LeDoux et al., 1983, 1986; Lin et al., 1984).

Although some striatal neurons respond to auditory or visual stimulation, those responding to somesthetic stimulation are much greater in number and occupy more striatal territory (Krauthamer, 1979; Lidsky et al., 1985; Reig and Silberberg, 2014). Furthermore, consistent with the role of somesthesis in behavioral habits such as grooming and whisking, the DLS in rats is concentrated with neurons that respond to both passive and active movements of the limbs and whiskers (Carelli and West, 1991; Brown, 1992; Mowery et al., 2011; Pidoux et al., 2011; Hawking and Gerdjikov, 2013; Reig and Silberberg, 2014).

## Neural Basis of Stimulus-Induced Responses in DLS

Neurons in rodent DLS respond to sensory stimulation, but the neural basis for these responses has not been resolved. Most projections to DLS originate from sensorimotor cortex (Brown et al., 1998; Alloway et al., 2006; Smith et al., 2012), and this fact is partly responsible for the widespread view that sensory-evoked responses in DLS are due entirely to corticostriatal inputs. This view, however, is not supported by experiments in which extracellular discharges were recorded simultaneously in DLS and SI of lightly-anesthetized rats (Mowery et al., 2011).

### Comparison of DLS and SI Response Properties

**Figure 5:**
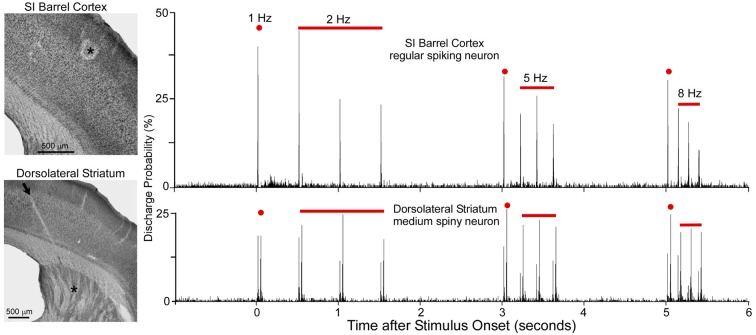
Neuronal responses recorded simultaneously in primary somatosensory cortex (SI; top) and DLS (bottom) during computer-controlled whisker deflections administered at 2, 5 and 8-Hz. Nearly two dozen whiskers were deflected in tandem in which each deflection consisted of back-and-forth movements in a 50-ms period. Photomicrographs illustrate the recording sites (asterisks) in SI and DLS. Peristimulus time histograms show that SI neurons adapt rapidly to repetitive whisker movements whereas DLS neurons adapt slowly. First deflection in each epoch is classified as 1-Hz because it is separated from previous deflections by at least 1 s. Adapted from Mowery et al. (2011).

As shown by Figure 5, when multiple whiskers are mechanically deflected in tandem, regular-spiking neurons in SI barrel cortex habituate quickly to repetitive whisker stimulation, but MSNs in DLS adapt very slowly (Mowery et al., 2011). Furthermore, whereas DLS neurons display high-fidelity “doublet” discharges in response to rapid back-and-forth whisker deflections, neurons in SI respond only to the initial phase of the same back-and-forth whisker motion.

Rapid adaptation is a classic feature of regular-spiking neurons in rodent SI cortex (Simons and Carvell, 1989; Ahissar et al., 2001; Khatri et al., 2004; Melzer et al., 2006; Chakrabarti and Alloway, 2009). This phenomenon is related to the fact that subthreshold responses in pyramidal neurons are characterized by EPSP-IPSP sequences in which an initial excitation is followed by prolonged inhibition (Innocenti and Manzoni, 1972; Creutzfeldt et al., 1974; Carvell and Simons, 1988; Moore and Nelson, 1998). Long-lasting cortical inhibition, which is mediated by local feed-forward and recurrent inhibitory circuits (Alloway et al., 1989; Swadlow, 2003), reduces the responsiveness of regular-spiking neurons when sequential sensory inputs are separated by short time intervals (Laskin and Spencer, 1979). Furthermore, corticostriatal neurons in layer V have intrinsic biophysical properties that cause them to adapt much more rapidly than other cortical neurons (Hattox and Nelson, 2007). These findings raise serious doubts about the possibility that rapidly-adapting corticostriatal neurons are capable of driving the slowly-adapting responses that have been observed in DLS (Mowery et al., 2011).

**Figure 6:**
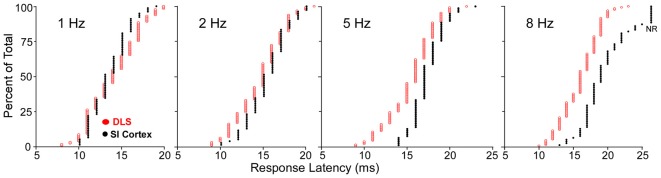
Cumulative distributions of response latencies of regular spiking and medium spiny neurons (MSNs) recorded, respectively, in SI and DLS during whisker deflections at different frequencies. At 8-Hz, nonresponsive neurons in SI are depicted by NR. Adapted from Mowery et al. (2011).

Comparisons of stimulus-induced response latencies in SI and DLS add further support to the view that SI cortex is not the only source of sensory inputs to DLS. As shown by cumulative latency distributions in Figure 6, DLS and SI neurons respond at the same time during low frequency whisker stimulation (1 or 2 Hz), but the SI neurons discharged after DLS neurons when the whiskers were deflected at higher frequencies (5 or 8 Hz). The DLS latency distributions were nearly identical at each tested frequency, but the distributions for SI were characterized by a shift towards longer latencies as stimulus frequency increased. This finding is consistent with the fact that prolonged cortical inhibition evoked by one stimulus will interfere with responses to subsequent stimuli that occur within intervals of 200 ms or less.

Some reports, however, have argued that stimulus-induced responses in DLS are determined by corticostriatal inputs. This view is based on studies in which whisker-evoked responses in DLS and SI cortex were recorded simultaneously in deeply-anesthetized animals (Pidoux et al., 2011; Hawking and Gerdjikov, 2013; Reig and Silberberg, 2014, 2016). In one study, for example, whole cell subthreshold recordings in DLS were conducted simultaneously with extracellular recordings in SI cortex during whisker stimulation of deeply anesthetized mice (Reig and Silberberg, 2014). In response to whisker movements evoked by air puffs, SI neurons discharged action potentials on a regular basis, but neuronal discharges were infrequently observed in DLS. The relative lack of discharges in DLS is not surprising because deep anesthesia suppresses the production of stimulus-evoked discharges in the striatum (West, 1998; Pidoux et al., 2011; Hawking and Gerdjikov, 2013). Consequently, subthreshold EPSPs were recorded to detect any stimulus-induced sensory input to DLS. Using this approach, Reig and Silberberg observed subthreshold responses in DLS immediately after the whisker-evoked neuronal discharges in SI. Based on this finding, they concluded that “responses in striatal neurons under our experimental conditions are generated primarily by cortical inputs without engaging a thalamostriatal shortcut (Mowery et al., 2011)”.

While this conclusion is valid for subthreshold responses recorded during deep anesthesia, several studies indicate that spontaneous and stimulus-induced activity in POm and DLS are suppressed by anesthesia or other manipulations that alter behavioral state (Carelli et al., 1997; West, 1998; Trageser et al., 2006; Masri et al., 2008). Stimulus-induced responses in POm are suppressed by GABAergic projections from ventral ZI (ZIv; Trageser and Keller, 2004; Lavallée et al., 2005), and this is significant because GABAergic transmission in the thalamus is enhanced by anesthesia (Detsch et al., 2002; Franks, 2008; Ying et al., 2009; Joksovic and Todorovic, 2010). These and other findings have prompted the view that POm transmits sensory information only in the awake state (Trageser et al., 2006; Urbain and Deschênes, 2007). However, if rats are maintained in a lightly-anesthetized state, as indicated by electrocorticographic (ECoG) activity ranging from 4 Hz to 6 Hz (Friedberg et al., 1999), we have consistently found that deflections of multiple whiskers will evoke neuronal discharges in both POm and DLS (Mowery et al., 2011; Smith et al., 2012).

**Figure 7:**
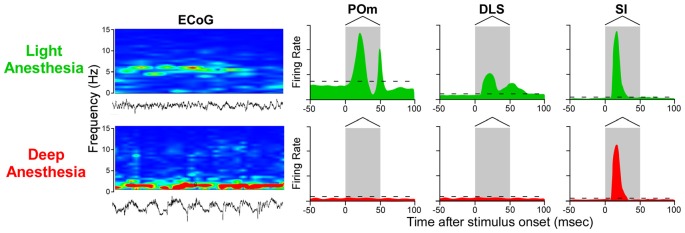
Stimulus-induced responses in POm, DLS and SI cortex as a function of behavioral state. In the lightly-anesthetized state, when electrocorticographic (ECoG) activity is 4–6 Hz, neurons in POm, DLS and SI display strong phasic responses to back-and-forth whisker deflections. When ECoG activity is dominated by frequencies below 1 Hz, however, neurons in POm and DLS are unresponsive, but neurons in SI cortex still display rapidly-adapting responses. Responses in the idealized peristimulus time histograms (PSTHs) are based on studies that examined the effects of anesthesia and other behavioral states on somesthetic responses in POm (Trageser et al., 2006; Masri et al., 2008; Smith et al., 2012; Alloway et al., 2014; Watson et al., 2015), DLS (West, 1998; Mowery et al., 2011; Smith et al., 2012; Alloway et al., 2014) and SI cortex (Chapin and Lin, 1984; Mowery et al., 2011).

As indicated by Figure 7, neurons in POm and DLS respond to whisker deflections in the awake or lightly-anesthetized state, but become unresponsive during deep anesthesia. By contrast, SI neurons respond robustly to peripheral stimulation in both the awake and the deeply-anesthetized state, which is consistent with cortical mapping studies performed during deep anesthesia (Chapin and Lin, 1984). Although time-locked neuronal discharges and subthreshold responses can be detected in SI and DLS, respectively, during deep anesthesia (Pidoux et al., 2011; Reig and Silberberg, 2014), the loss of stimulus-induced DLS discharges during deep anesthesia is correlated with a similar loss of responsiveness in POm.

### Comparison of Stimulus-Induced Responses in POm and DLS

Simultaneous extracellular recordings of neuronal discharges in POm and DLS of lightly-anesthetized rats have revealed many similarities in the stimulus-induced responses in these two regions. In contrast to the rapid habituation observed in SI, neurons in POm and DLS display slowly-adapting responses during repetitive whisker stimulation (Mowery et al., 2011; Smith et al., 2012; Alloway et al., 2014). In fact, rapid back-and-forth whisker movements evoke “doublet” responses in both POm and DLS, but not in SI cortex. Furthermore, when POm and DLS neurons are recorded simultaneously during whisker stimulation, POm discharges consistently precede the DLS discharges by 1–2 ms regardless of stimulus frequency (Smith et al., 2012).

Several reports indicate that POm and DLS neurons have relatively complex response properties when compared to neurons in VPM or SI cortex. Neurons in VPM and SI barrel cortex are easily activated by deflections of a single whisker (Brumberg et al., 1996), but whisker-sensitive neurons in POm have larger receptive fields and are less responsive unless multiple whiskers are stimulated simultaneously (Chiaia et al., 1991b; Diamond et al., 1992; Sosnik et al., 2001). Similarly, DLS neurons may occasionally discharge in response to the motion of a single whisker, but most whisker-sensitive neurons in DLS require stimulation of multiple whiskers (Carelli and West, 1991; Mowery et al., 2011). Even in the awake state, neuronal discharges are not consistently evoked in DLS when only a single whisker is deflected (Sippy et al., 2015). When these facts are considered together, the relatively crude somatotopic organization and the prevalence of neurons with large receptive fields emphasizes the importance of global, spatially-distributed sensory stimulation for activating both POm and DLS.

## Functions of the Thalamostriatal Projections from POm and Pf

Neurons in both Pf and POm respond to sensory inputs, but the stimulus-induced responses in these nuclei are significantly different. The CM/Pf complex receives multimodal sensory inputs from the intermediate and deep layers of the superior colliculus (Yamasaki et al., 1986; Nothias et al., 1988; Grunwerg and Krauthamer, 1992; Krout et al., 2001; Coizet et al., 2007; Schulz et al., 2009; Alloway et al., 2014), which process visual, auditory and somatosensory inputs (May, 2006). Consistent with this, recordings in both primates and rats have shown that neurons in the CM/Pf complex display multisensory responses, but these responses habituate very quickly during repeated stimulus presentations (Chiaia et al., 1991b; Grunwerg and Krauthamer, 1992; Matsumoto et al., 2001; Minamimoto and Kimura, 2002).

By contrast, POm receives very few inputs from the superior colliculus (Roger and Cadusseau, 1984), and the relative lack of auditory and visual inputs has prompted the view that POm is mainly concerned with processing somesthetic information (Diamond, 1995). Furthermore, POm neurons adapt very slowly to repetitive whisker stimulation (Sosnik et al., 2001; Smith et al., 2012; Alloway et al., 2014), and simultaneous recordings in Pf and POm indicate that response latencies are significantly shorter in POm than in Pf (Alloway et al., 2014). These facts are consistent with reports showing that POm receives somesthetic inputs directly from the trigeminal nuclei (Chiaia et al., 1991a,b; Veinante et al., 2000; Sosnik et al., 2001; Masri et al., 2008), whereas Pf depends on sensory information that is transmitted across longer multisynaptic routes that depend on inputs from the superior colliculus (see Figure 2).

### Collicular Control of Pf and POm

The superior colliculus is known for having extensive connections with brainstem circuits that control eye movements and related aspects of visual orientation so that attention can be re-directed to unexpected, highly salient stimuli (Sparks, 1986; May, 2006). Consistent with these functions, the thalamostriatal projections from POm and Pf appear to be part of a feed-forward network that regulates communication between the superior colliculus and the basal ganglia.

Tracing studies indicate that superior colliculus projects to Pf and other intralaminar thalamic nuclei (Yamasaki et al., 1986; Yamasaki and Krauthamer, 1990; Krout et al., 2001; Alloway et al., 2014). In addition, the superior colliculus sends dense projections to ZIv, which is located immediately dorsal to the STN (Roger and Cadusseau, 1985; Mitrofanis, 2005; Alloway et al., 2014; Watson et al., 2015; Kita et al., 2016). The ZIv is notable because it is concentrated with GABAergic neurons that project to POm (Barthó et al., 2002; Watson et al., 2015), and the inhibitory influence of ZIv on POm was demonstrated by showing that ZI lesions enhance somesthetic responsiveness in POm (Trageser and Keller, 2004; Lavallée et al., 2005).

**Figure 8:**
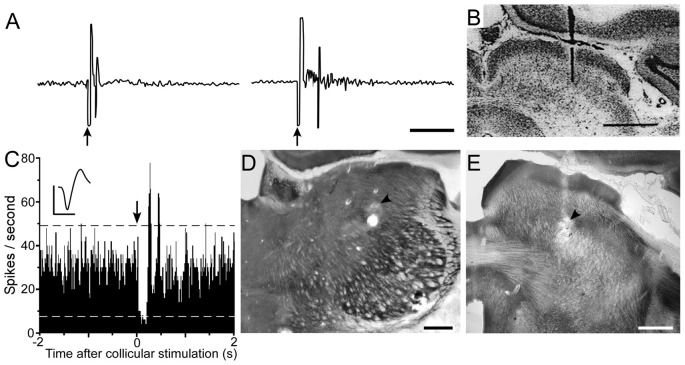
Electrical stimulation of the superior colliculus exerts opposing influences on Pf and POm. **(A)** Excitatory responses recorded in the Pf and centrolateral nuclei following electrical stimulation (arrows) of the superior colliculus. **(B)** Histology shows location of bipolar stimulating electrode in superior colliculus. Data reprinted with permission (Grunwerg and Krauthamer, 1992). Scale Bars: 10 ms **(A)**, 1 mm **(B)**. **(C)** PSTH shows inhibition of POm activity following electrical stimulation (arrow) of superior colliculus. Waveform scales: 1 ms, 100 μV. PSTH: 50 trials, 5-ms binwidths. **(D,E)** Photomicrographs depicting recording and stimulating sites (arrowheads) in POm and superior colliculus. Adapted from Watson et al. (2015).

According to the circuit connections in Figure 2, activation of the superior colliculus should exert opposing influences on Pf and POm, both of which project to the striatum. As indicated by Figure 8, prior work has shown that Pf and other thalamic intralaminar neurons are activated by the superior colliculus (Grunwerg and Krauthamer, 1992). Consistent with the intervening inhibitory projections from ZIv to POm, Figure 8 also demonstrates that the superior colliculus can inhibit spontaneous activity in caudal POm (Watson et al., 2015). Furthermore, in contrast to the relatively brief activation of Pf that is typically evoked by collicular stimulation, neuronal activity in POm is inhibited by the superior colliculus for a prolonged period.

## Opposing Behavioral Functions of Pf and POm Striatal Projections

The differential influences exerted by the superior colliculus on Pf and POm suggest that the thalamostriatal projections from these nuclei have behavioral functions that are incompatible with each other. Although the precise nature of these behavioral functions remain unclear, the available data suggest that the CM/Pf complex has a role in facilitating corticostriatal activation of the indirect pathway (Ding et al., 2010), whereas the POm nucleus is involved in facilitating the functions of the direct pathway.

Thalamostriatal projections from the CM/Pf complex innervate all of the dorsal striatum and appear to be involved in gating the influence of corticostriatal projections throughout this region. In addition to contacting MSNs, these projections provide strong input to cholinergic interneurons (Meredith and Wouterlood, 1990; Lapper and Bolam, 1992; for review, see Smith et al., 2014). In brain slices, electrical stimulation of the CM/Pf complex evokes a burst of activity among cholinergic interneurons that is followed by a noticeable pause (Ding et al., 2010; Schulz and Reynolds, 2013; Doig et al., 2014). This burst-pause sequence among cholinergic interneurons is coupled to an immediate decrease in activity among all MSNs, which is followed by a selective increase in the sensitivity of the indirect pathway to corticostriatal inputs (Ding et al., 2010). By contrast, inhibition of the cholinergic interneurons reduces catalepsy and other motor deficits in a mouse model of PD (Maurice et al., 2015). Furthermore, these behavioral effects are associated with prolonged inhibition of the substantia nigra pars reticulata (SNR), which suggests increased output of the direct pathway.

Converging evidence indicates that the indirect pathway has a suppressive effect on behavior, whereas the direct pathway facilitates motor output (Albin et al., 1989; DeLong, 1990; Kravitz et al., 2010; Sippy et al., 2015). In view of data showing that the CM/Pf complex is activated by unexpected stimuli that cause a shift in attention (Matsumoto et al., 2001; Minamimoto and Kimura, 2002), this thalamic region appears to be involved in suppressing an on-going behavior so that a more adaptive behavior can be selected (Ding et al., 2010; Thorn and Graybiel, 2010).

In contrast to the CM/Pf complex, the POm nucleus innervates the DLS but no other part of the striatum (Smith et al., 2012; Alloway et al., 2014). Given the importance of DLS for expressing habitual behaviors in a familiar context (Cromwell and Berridge, 1996; Aldridge and Berridge, 1998; Yin et al., 2004; Yin and Knowlton, 2006; Yin, 2010), POm projections to DLS appear to be vital for transmitting somesthetic information that is critical for executing specific sequences of well-learned behaviors. To the extent that CM/Pf activation initiates the circuit mechanisms that pause an ongoing behavior, any role played by POm in facilitating a DLS-mediated sensorimotor habit is likely to be incompatible with the functional role of the CM/Pf complex. Therefore, consistent with the physiological evidence, collicular-induced activation of Pf should be accompanied by inhibition of POm to ensure that an ongoing habitual behavior is suppressed when an unexpected stimulus requires selection of another behavior.

### Unresolved Issues Concerning the Functional Role of POm

The available evidence supports the hypothesis that POm transmits sensory-related information to DLS to facilitate the expression of behavioral habits, but several critical issues remain unresolved. No studies have characterized POm neuronal activity during the performance of a behavioral task, nor has there been any attempt to characterize whether POm responses are altered as a behavior is transformed into a habit. Related to this functional role of POm, no study has determined whether POm impairment blocks the acquisition or expression of a well-learned behavioral habit.

A related issue concerns how thalamostriatal inputs from POm are coordinated with respect to neuronal responses in DLS, and to what extent POm cooperates with sensorimotor cortex in activating DLS, either in response to sensory stimulation or with respect to the acquisition of a behavioral habit. Addressing this latter issue is difficult, however, and would require chronic, simultaneous recordings from all three brain regions during prolonged behavioral training.

Another issue concerns the specific mechanisms that determine whether POm is activated by sensory inputs or is inhibited by stimulus-induced activation of the superior colliculus. Both POm and the superior colliculus receive somesthetic information directly from the trigeminal nuclei (see Figure 2), and these circuits could either activate POm or activate the collicular-incertal pathway cause inhibition of POm. The nature of the somesthetic inputs and the circuit mechanisms that determine whether POm is activated or inhibited represent an important problem for understanding the behavioral functions of POm and its thalamostriatal projections. Several findings indicate that POm is inhibited by ZIv (Trageser and Keller, 2004; Lavallée et al., 2005; Watson et al., 2015), and while some have suggested that MI projections to ZI may release POm from incertal inhibition during a behavioral activity (Urbain and Deschênes, 2007), evidence for this circuit mechanism has not been obtained.

## Clinical Significance of Thalamostriatal Projections from POm

**Figure 9:**
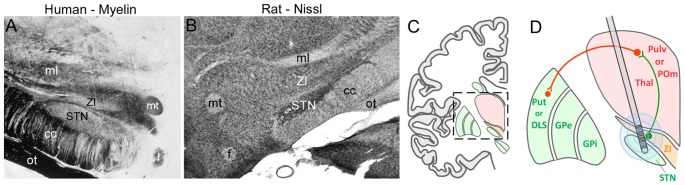
Deep brain stimulation (DBS) in the STN can alter ZI activity. **(A,B)** Coronal sections from human and rat brains illustrate the close proximity of STN and ZI in myelin and Nissl-processed sections, respectively. **(C)** Schematic of a coronal section through the human brain shows the diencephalon with respect to the basal ganglia. Inset shows the region depicted in panel **(D)**. **(D)** Schematic showing how multiple electrode contacts combined with current spread may cause stimulation of both STN and ZI in both the primate and rodent brain. Green lines, inhibitory connections; red lines, excitatory connections. Abbreviations: GPe, globus pallidus external; GPi, globus pallidus internal; POm, posteromedial nucleus; Pulv, Pulvinar; Put, Putamen; STN, subthalamic nucleus; Thal, thalamus; ZI, zona incerta.

The ZI is located directly above the STN, which is a major target of DBS used to treat PD. Given that STN and ZI are adjacent to each other (see Figure 9), it is not surprising that electrode contacts intended for STN have often led to inadvertent stimulation of ZI. Many clinicians report that stimulation of caudal ZI (ZIc) reduces PD symptoms (as measured by scores on the Unified Parkinson’s Disease Rating Scales), lowers the L-dopa dosage needed to treat PD, and is effective in treating essential tremor (Plaha et al., 2006, 2008; Sun et al., 2008; Blomstedt et al., 2011, 2012; Fytagoridis et al., 2014; Lukins et al., 2014). Furthermore, the optimal DBS target for treating PD is at the interface of the ZIc and STN (Caire et al., 2013; Garcia-Garcia et al., 2016), presumably because both regions are co-stimulated at that location. Although the mechanisms of DBS are controversial, some data indicate that high-frequency stimulation disrupts the timing of aberrant oscillatory signals in STN and other sites (Vitek, 2008; Ponce and Lozano, 2010; Agnesi et al., 2013; Miocinovic et al., 2013).

The circuit connections of ZI are phylogenetically conserved in mammalian brain. In both rodents and primates, superior colliculus innervates Pf and ZIv, and the latter structure is concentrated with GABAergic neurons (Mitrofanis, 2005; Watson et al., 2014). While ZIc has been implicated with the beneficial effects of DBS, all sectors of ZI are interconnected (Power and Mitrofanis, 1999), and it is likely that ZIc stimulation would also influence GABAergic neurons located more rostrally in ZI. If DBS of human ZIc alters the activity of the GABAergic projections to the human homolog of POm, then an increase in the activity of its thalamostriatal projections could facilitate the striatal mechanisms that enable execution of normal behavioral movements.

Although evidence is limited, data from several sources suggest that the anterior pulvinar nucleus represents the primate homolog of rodent POm (Butler and Hodos, 2005). The primate pulvinar adjoins the dorsomedial side of the ventrobasal complex, just as POm does in rodents (Jones, 1985; Pons and Kaas, 1985). Both the pulvinar and POm have corticothalamic and corticocortical connections that signify higher-order thalamic nuclei (Feig and Harting, 1998; Killackey and Sherman, 2003; Alloway et al., 2008), and the divergent collateral projections from rodent POm to DLS and SI cortex resemble the collateral projections from the pulvinar to putamen and SI cortex in primates and other species in related phylogenetic lineages (Lin et al., 1984; Jones, 1985; Pons and Kaas, 1985; Smith and Parent, 1986; Day-Brown et al., 2010). The primate pulvinar remains poorly understood, however, and investigations of the collicular-incertal-POm-DLS circuit in the rat, and eventually in primates, could represent useful models for determining why ZI stimulation is beneficial for treating PD and essential tremor. Hopefully, elucidation of this mechanism would reveal new therapeutic approaches in the treatment of PD and related neurological diseases.

## Author Contributions

KDA wrote the draft of the manuscript, constructed some figures and reviewed the literature described in this article, and approves this submission and takes responsibility for all information contained in this article. JBS made significant revisions to multiple drafts of this review article, constructed some figures and reviewed the literature cited in this article, and approves this submission, and takes responsibility for all information contained in this article. TMM made significant revisions to multiple drafts of this article, and contributed to the cited literature, and approves this submission, and takes responsibility for all information contained in this article. GDRW made significant revisions to multiple drafts of this article, and constructed some of the figures, and approves this submission, and takes responsibility for all information contained in this article.

## Conflict of Interest Statement

The authors declare that the research was conducted in the absence of any commercial or financial relationships that could be construed as a potential conflict of interest. The handling Editor declared a shared affiliation, though no other collaboration, with one of the authors with one of the authors GDRW and states that the process nevertheless met the standards of a fair and objective review.
